# The FHJ debate: Will artificial intelligence replace clinical decision making within our lifetimes?

**DOI:** 10.1016/j.fhj.2024.100178

**Published:** 2024-09-19

**Authors:** Joshua Hatherley, Anne Kinderlerer, Jens Christian Bjerring, Lauritz Aastrup Munch, Lynsey Threlfall

**Affiliations:** a Aarhus University, Department of Philosophy and History of Ideas, Denmark; b Royal College of Physicians Clinical Digital Lead, London; c Royal Victoria Infirmary Newcastle, Newcastle University - BRC, England

## Proposition: AI systems will replace clinical decision making within our lifetimes

*Joshua Hatherley, Jens Christian Bjerring and Lauritz Aastrup Munch*

AI systems could replace clinical decision making in two ways. First, from the ‘top down’, by way of hospitals and healthcare organisations. Second, from the ‘bottom up’. by way of patients and patient-facing AI systems. In what follows, we argue that AI systems are likely to replace clinical decision making within our lifetimes – in both a top-down and bottom-up fashion – for several institutional, economic and psychological reasons.

## Top-down

Top-down replacement occurs when hospitals and healthcare organisations reduce the degree of human involvement, authority or discretion in clinical decision making tasks to the extent that human judgement becomes subordinate to, or replaced with, AI-driven reasoning. It is likely to occur within our lifetimes for three reasons.

First, hospitals and healthcare organisations are facing increasing pressure to increase productivity and economic efficiency to address both national and global health challenges, including population growth and increasing rates of chronic illness. Purchasing and maintaining AI systems will often be cheaper than ongoing employment for human professionals since AI systems can process clinical cases far more swiftly and efficiently than human clinicians,^1^ and they can do so without becoming fatigued or requiring breaks for rest and relaxation. Moreover, AI systems cannot unionise or demand better working conditions. In a time where hospitals are increasingly squeezed for time and money, many are therefore likely to prioritise investing in technology over investing in humans.

Second, as AI becomes increasingly accepted in medicine, top-down replacement provides hospitals, healthcare organisations and AI organisations with a compelling opportunity to maximise their return on investment. Many AI systems have demonstrated impressive performance in a wide range of clinical tasks.^2^^,^^3^ In some cases, AI systems even appear to match or exceed the performance of human clinicians.^4^^,^^5^ However, the quality of data contained in repositories used to train medical AI systems – such as electronic health records – is highly inconsistent.^6^ Primarily, this is due to errors, inconsistencies and oversights in clinicians’ data-recording practices. By reducing human involvement and authority over the clinical decision-making process, healthcare organisations and AI developers can improve the quality of these datasets by standardising data collection processes, thereby boosting the overall performance of medical AI systems.

Third, AI and healthcare organisations are strongly incentivised to expand their power and prestige,^7^^,^^8^ and top-down replacement is a compelling strategy for achieving this. For instance, by reducing human involvement in clinical decision making, AI organisations can increase their capacity to monitor, manipulate and control patients’ behaviour for economic gain, otherwise known as their ‘instrumentarian’ power.^9^ Moreover, new medical technologies increase the power and prestige of institutions that choose to adopt them,^10^^,^^11^ and medical AI systems are currently subject to an enormous degree of hype and attention. Pundits anticipate that medical AI will deliver a range of benefits that include improved patient health and wellbeing, enhanced health equity, and increased patient safety.^3^^,^^12^^,^^13^. Regulatory approvals of medical AI systems in the USA have also rapidly accelerated since around 2018^14^, and the McKinsey Institute has even estimated that AI technologies will generate between US$200–$300 billion of value in the healthcare sector.^15^

Of course, professional and regulatory bodies are likely to persist in their recent attempts to restrict AI systems to a merely supportive role in medicine.^16^ Ultimately, however, this project is unlikely to succeed, given that AI systems are likely to increasingly outperform human clinicians in the near to mid-term future. Moreover, effectively combining human reasoning with AI reasoning is notoriously difficult due to a broad range of human factors challenges including automation bias, algorithmic aversion and automation complacency.^17^, ^18^, ^19^ Eventually, therefore, hospitals and healthcare organisations are likely to pursue top-down replacement to overcome or avoid such challenges.

## Bottom-up

Bottom-up replacement occurs when patients themselves minimise the degree of human involvement in clinical decision making by turning increasingly to AI systems, rather than human clinicians, for medical advice. It is likely to occur during our lifetimes for three reasons.

First, healthcare is becoming increasingly expensive and unaffordable, particularly for patients from low- and middle-income backgrounds, and patients in countries without universal health coverage. Citizens worldwide are also facing increasing financial pressures due to inflation, stagnant wages and the economic fallout of COVID-19. By seeking out medical advice from AI systems rather than human clinicians, patients can substantially reduce the financial pressures associated with healthcare. Indeed, many patients already report self-diagnosing with large language models such as ChatGPT.^20^^,^^21^

Second, patients continue to be disempowered and disconnected from their own medical care and treatment due to imbalanced power dynamics in clinician–patient relationships.^22^ However, bottom-up replacement provides patients with an opportunity for greater empowerment and control in healthcare. AI systems, in combination with other technologies such as wearables, are likely to enable patients to manage many aspects of their health independently of human clinicians and reduce patients’ reliance on frequent visits to human clinicians for routine check-ups and in-person consultations.^23^ Indeed, the trend towards self-monitoring with wearables is already strong, and it is likely to be accelerated by AI.^24^ Additionally, the data collected by wearables can be shared with other AI systems – as well as human practitioners, of course – which is likely to improve the quality of AI-assisted care and decision making over time. Relatedly, since AI systems can analyse and remember vast amounts of data, the level of personalisation that these systems can achieve is likely to exceed what busy human clinicians can provide in the near- to mid-term future.

Third, many patients will likely even come to *prefer* receiving healthcare services from AI systems rather than human clinicians. AI systems can simulate care, and can in some ways do so better than human clinicians.^25^ Empirical studies have already shown that users are more comfortable revealing sensitive or potentially embarrassing information to machines rather than human professionals.^26^^,^^27^ Patients have also been found to interpret text that is generated by large language models as more empathetic and caring than text written by human clinicians.^28^ While medical AI systems cannot *actually* care for or empathise with patients – at least, not for the foreseeable future – the strong tendency for humans to anthropomorphise AI systems suggests that this does not matter. As we have already seen with therapeutic robots like Paro, patients need not *actually* be cared for in order to *feel* cared for.^29^

## Objections

Our opponents raise several objections against the preceding arguments. First, they suggest that AI systems are unlikely to achieve the degree of safety or flexibility needed to replace *all* parts of the clinical decision-making process. One key reason for this is that these systems cannot incorporate contextual information or information about patients’ values and preferences into their reasoning processes. Another reason is that clinical decision making goes beyond simple pattern recognition; it involves genuinely engaging with patients in a collaborative decision-making process that AI systems cannot currently replicate.

However, many of these limitations are likely to be addressed as the technology improves. Indeed, multimodal systems are already advancing to learn from facial expressions, tones of voice, and more.^30^ AI systems are increasingly being designed to incorporate patient-specific data, including medical history, genetic information and lifestyle factors, in their reasoning.^31^ Patient interactions with AI systems could be structured to allow for input of preferences and values, which can then be factored into the AI's decision-making process.^32^ Moreover, the objection relies on an overly stringent definition of ‘replacement’ in which human clinicians no longer play *any* role in clinical decision making. We agree that humans may continue to play a role in clinical decision making, but we suggest that their authority and responsibilities will diminish with the progression of AI to the point that human clinicians are at least subordinated to these systems. Despite any continued involvement of human clinicians, therefore, it will ultimately be the AI systems that are in command.

Second, our opponents highlight that human–AI teams appear to perform better than AI systems or humans alone. Relatedly, they argue that recent performance estimates of AI systems are likely exaggerated in practice due to human factors challenges such as human error, algorithmic bias and automation complacency. We agree that clinician–AI teams may reliably outperform medical AI systems operating alone. We also agree that current performance estimates for medical AI systems may be inflated due to several human factors challenges. As noted above, however, medical AI systems need not be completely autonomous to effectively replace human clinicians in clinical decision making, and we suggest that the balance of authority in clinician–AI teams will increasingly shift toward AI systems. Indeed, one reason that hospitals and healthcare organisations will be incentivised to pursue a minotaur-based approach to clinician–AI teams is precisely to avoid these human factors challenges, as we also emphasised earlier.

Finally, our opponents argue that top-down and bottom-up replacement are unlikely to occur because clinicians and patients are unlikely to trust AI systems that are known to contain biases, generate inexplicable predictions, generate ‘bullshit’ outputs and hallucinate information. Yet, these weaknesses in medical AI systems are gradually being addressed through technical methods (eg explainability methods, improved data curation and algorithmic auditing), and their associated risks are likely to diminish as the technology improves. The integration of AI into clinical decision making will not result in overnight replacement, but is rather a gradual, iterative process. As AI systems demonstrate increased accuracy and reliability over time, both clinicians and patients will likely build more trust in these technologies.

Ultimately, therefore, we find these objections serious, but unconvincing.

## Conclusion

AI systems are likely to replace clinical decision making within our lifetimes, both from the top down and the bottom up. This is not an inevitable future, nor is it necessarily a desirable one. At present, however, without serious and radical change to our health systems and technological governance and regulations, it is likely to materialise in response to a variety of institutional, economic and psychological challenges that currently affect patients, hospitals and healthcare systems at large.

## Opposition: AI will not replace clinical decision making in our lifetime

*Anne Kinderlerer and Lynsey Threlfall*

To answer the question, we will first define clinical decision making. Is it merely the process by which the clinician sifts available data and arrives at a diagnosis and a treatment which they communicate to the patient? Given the exponential growth in medical data – the NHS alone generates billions of clinical data points a week^33^ – it is easy to argue that at some point humans will be unable make decisions, as the human brain will be unable to assimilate the volume of data.^34^

We argue, however, that the task of clinical decision making is not merely pattern recognition based on available data, but that it encompasses the process in which patient and clinician arrive at a shared model of pathology and a treatment plan.

This is a fundamentally human method of care, as central to the clinician’s art as history taking, physical examination and patient education.^35^ Computer-based clinical decision support systems (CDSSs) cannot yet follow the multi-step process to collect, contextualise and integrate the clinical data and the biopsychosocial context of the individual patient so central to beneficial outcomes.^36^

CDSSs are ‘active knowledge systems which use two or more items of patient data to generate case-specific advice in more or less real time’.^37^ The proposers argue that CDSSs are expected to improve and ultimately replace clinical decision making by making it faster, cheaper and less prone to error. Certainly, current algorithms can process large volume data more swiftly than humans, but several studies have demonstrated that the most effective results come from AI performing the initial screen with humans reviewing the more complex cases.^38^^,^^39^ AI has been shown to have impressive results matching or improving upon clinicians in areas where there are clear guidelines and widely accepted evidence.^40^, ^41^, ^42^ However, Hager *et al* suggest that large language models (LLMs) cannot apply that knowledge to more realistic clinical scenarios encompassing the complexity of patients in the real world,^43^ contrasting with the performance on medical exams cited by the proposers. In an increasingly ageing population with multiple long-term conditions, it is difficult to see AI replacing humans.

There is reason to think that the difference in the way that AI models and human beings ‘think’ raises questions about the ability of such systems to operate autonomously. Data-driven CDSSs using big data sets generate conclusions that are not easy to explain.^44^ And if, as the proposers celebrate, we develop ‘AI that begets AI’, then the algorithms will become increasingly complex, further worsening the problem of explainability.^45^ There are already concerns about the black-box nature of existing decision support systems.^46^^,^^47^ This lack of explainability affects the trust of both clinicians and patients in clinical decisions. Lack of trust affects the likelihood of the patient accepting decisions about treatment, and potentially infringes their autonomy to make choices.^48^ Furthermore, this lack of explainability means that it is more difficult to identify bias in the outcomes of AI decision making. Bias is arguably more and not less likely in these systems.^49^ Just as our concepts of disease and decisions on treatment are value-laden and culturally specific,^50^ data used to train LLM encode individual and societal values, which may differ from those of the physician and patient in the room and which will not necessarily be transparent.^51^

As LLMs interact with the world primarily through language, as humans do, the hope is that they are poised to be the point of access to the AI solutions of the future. However, there are moral and practical complexities with the way they function. It is worth reflecting on the relationship of the output of an LLM, such as ChatGPT, to truth. They are designed with no reference to truth, but only to the plausibility of the next word in the sequence: to be plausible rather than correct. There is no difference in model function between ‘truthful’ outputs of the LLM and times when it ‘hallucinates’. In this sense the overall activity of LLM, is better understood as *bullshit* in the sense explored by Frankfurt.^52^ It is therefore not surprising that current LLMs are hasty and unsafe clinical decision makers.^43^

Wider society is likely to reject potentially unsafe algorithms where bias is both baked in and undetectable, and such systems are unlikely to add to the prestige of organisations that deploy them.

AI is a disruptive technology and, as with all disruptive technologies, we agree with the proposers that it will democratise healthcare and give patients options to manage their healthcare better and more cheaply.^53^ Wearable technologies are improving continuously, and AI algorithms can already diagnose hypertension, left ventricular disfunction and sleep apnoea using data from our smart watches.^54^^,^^55^ This will change the conversations that patients are able to have with their human clinicians. Studies indicate that most patients are willing to use AI for self-diagnosis,^56^ as they already do with search engines. But as we have argued, AI cannot integrate patient preferences, laboratory data, response to treatment and guidelines into a personalised treatment plan.

Even if, as the proposers of the motion argue, it were possible to get beyond these fundamental problems in design that limit the building of an autonomous CDSS, experience of implementing technology into complex health systems does not suggest that it will be possible to build widespread safe systems in our lifetime.^57^ This will limit the ability of healthcare organisations to impose such systems in order to save costs. Roll out of AI in place of humans in a ‘top-down’ replacement strategy requires significant ‘fixing’ of the underlying technological infrastructure. Health data to train algorithms and to deploy risk-based algorithms are siloed and poorly curated.^58^ Autonomous CDSS will need to be integrated into existing digital systems, which currently exist as multiple subsystems, increasing the difficulty of successful implementation with workable processes.^59^ Such implementation will require large upfront costs, not only to fix the technological issues but also in the human expertise required for deployment. Large-scale transformative digital projects have a history of failure, particularly in healthcare.^60^ Indeed, its complex nature makes the implementation of rule-based algorithms without human oversight even more difficult.^61^

The proposers argue for significant benefits of AI, that it is faster, cheaper, more accurate, better at learning and preferred by patients. However, to achieve these outcomes the technology must be deployed correctly. The idealised performance promised by the advocates of innovation doesn’t always marry up with the complex socio-technical interactions seen in the real world,^62^^,^^63^ as we have seen in the well-publicised failure of the autonomous computer system designed to operate without the possibility of human correction in the Post Office in the UK.^64^ We can already see that well-validated AI predictive models that appear accurate in development are increasingly found to be ineffective or unsafe when deployed in clinical pathways in real healthcare systems.^65^

In a future where clinical decision making is replaced by AI, who is creating and inputting the data? If humans are involved, there will still be workarounds, whether these are clinician or patient generated, and these will compromise the data integrity.^66^^,^^67^ The consequences of low-quality data in a sophisticated algorithm, when a human is merely observing the loop and not sense-checking, is that whole populations may be misdiagnosed or mistreated. Workarounds are prevalent in all industries wherever a human interacts with a machine; lack of high-quality training is often felt to play a part alongside a lack of understanding about the capability of the software.^68^ Within areas of high social deprivation, for whom the proposers argue there may be the greatest benefits, we often have adult populations with a reading age of 8–9 years old, populations for whom English may not be their first language, or who have additional learning needs. How can we ensure that these populations receive adequate training and education to interact in the desired way with AIs? Given that these programmes will be trained on data predominately from developed English-speaking countries, there is the risk of widening global health inequalities rather than, as the proposers argue, reducing them. There is significant risk of creating new hazards in systems without controls and checks able to identify the risk.

## Conclusions

Healthcare and its delivery are a ‘wicked’ problem – difficult to resolve because they are difficult to define – and it is easy to oversimplify such issues in the quest for ideal solutions.^69^ The proposers argue that the takeover of AI is inevitable, if not necessarily desirable, driven by social, economic and psychological imperatives.

We should not blindly walk toward a future where AI supersedes humans. Modern history is littered with examples of grandiose IT schemes that ultimately fail to deliver benefits. The autonomy of patients and clinicians is precious; let us not carelessly cast it aside. Fundamentally, do you want a ‘bullshit machine’ to oversee your healthcare?

What do you think? Vote at https://forms.office.com/e/eeHsZfa6vz until 18 November 2024. Do you like the debate feature? Send us your thoughts to fhj@rcp.ac.uk.

## CRediT authorship contribution statement

**Joshua Hatherley:** Conceptualization, Writing – original draft, Writing – review & editing. **Anne Kinderlerer:** Writing – original draft, Conceptualization. **Jens Christian Bjerring:** Conceptualization, Funding acquisition, Writing – review & editing. **Lauritz Aastrup Munch:** Conceptualization. **Lynsey Threlfall:** Writing – review & editing.

## Declaration of competing interest

All authors declare no competing interests.
